# Does a novel exergame challenge balance and activate muscles more than existing off-the-shelf exergames?

**DOI:** 10.1186/s12984-019-0628-3

**Published:** 2020-01-15

**Authors:** Jente Willaert, Aijse Willem De Vries, Julie Tavernier, Jaap H. Van Dieen, Ilse Jonkers, Sabine Verschueren

**Affiliations:** 1 KU Leuven (Faculteit bewegings- en revalidatiewetenschappen), Leuven, Belgium; 2 Vrije Universiteit Amsterdam (Faculteit gedrags- en bewegingswetenschappen), Amsterdam, The Netherlands

**Keywords:** Exergames, Balance, Elderly, Fall prevention

## Abstract

**Background:**

Novel balance-targeting exergames controlled with off-the-shelf hardware, were developed based on current recommendations for balance training in healthy older adults and documented shortcomings of existing games. The aim of this study was to explore the feasibility of these novel exergames as training tool for elderly and, more specifically whether these games can elicit more challenging weight shifts and higher levels of muscle activity compared to existing off-the-shelf exergames. Furthermore, the motivational pull in these new games was studied.

**Methods:**

Sixteen healthy older adults were recruited to play the novel games and two reference games that were found to be the most challenging ones in terms of weight shifts or muscle activity in previous studies. Weight shifts were expressed relative to participants’ Functional Limits of Stability (FLOS). Muscular challenge of the games was quantified by dividing the signal into 200 ms blocks and determining the average muscle activity within these blocks. The muscle activity was normalized to maximal voluntary contractions (MVC) to categorize the blocks in zones of < 40, 40–60, 60–80 and > 80% MVC. Subsequently, the number of blocks per intensity level and the number of consecutive blocks above 40% were determined. Motivation to play the games was assessed using the Intrinsic Motivation Inventory (IMI) and scores between the games were analyzed using Generalized Estimated Equations (GEE).

**Results:**

The novel exergames successfully elicited center of mass (COM) displacements with medians of around 80% of FLOS or higher for all directions. Furthermore, the COM displacements in the novel games were larger for each direction than in the reference games, although for one game the sideward left direction reached significance only at the third trial. Compared to the existing games, longer blocks of muscle activation above 40% MVC were found, but overall intensity remained low. IMI scores were high on all subscales, indicating that older adults experienced the games as motivating.

**Conclusion:**

We conclude that affordable hardware can be used to create challenging and enjoyable balance training programs using exergames. The exergames that were successful in eliciting challenging weight shifts and muscle activity should now be further studied in longitudinal randomized controlled interventions, to assess effects on balance, muscle strength and eventually fall risk in healthy older adults.

## Background

Studies report that 30–40% of people older than 65 will fall at least once per year and about 10–20% of these falls will result in hospitalization [1, 2]. The number of people aged 65 and older will increase due to the demographic developments worldwide, which will further increase the total number of falls [3]. Major risk factors for falling are an age-related decrease in functional capabilities, especially in balance control and muscle strength [4, 5]. Multidimensional training programs have been shown to ameliorate these risk factors and reduce fall risk in older adults. This is especially the case when strength training and sufficiently challenging balance exercises are provided for at least 3 h per week [1, 2, 6, 7]. However, ongoing participation in a training program is needed to prevent fading of the benefits due to the progressive strength and balance decline caused by aging [2, 6]. As long-term, structural supervised training is costly, home-based training appears most promising for long-term effects. Sadly, adherence to traditional home-based training programs is low due to the repetitive nature of the exercises, lack of perceived usefulness and therefore motivation [8, 9].

The use of computer games to aid in balance training for older adults, also called exergames balance training, receives increasing attention [10–12]. In this study, exergames are defined as computer games using commercial consoles as the Wii and the Kinect console and that are controlled with body movements. Different commercial games are already available that might have a balance training potential [10–12]. Potential benefits of exergames over conventional training are: an increase in motivation and thereby adherence [13], the option to offer dual task training [14], the option to provide different forms of feedback [15] and to adapt the training intensity to the skill level of the player so that individualized progression is possible. However, the latter is not always possible in commercial games. Despite these promising features, systematic reviews report varying results on balance [10–12], possibly due to the wide variability in games that have been studied and the fact that these games were not specifically developed with the aim to improve balance in older adults. In conventional balance training, strength and specific balance training were shown to be key elements in preventing falls [2, 6, 16, 17]. It is recommended that balance training is sufficiently challenging by requiring weight-shifts to the limits of stability, by reducingthe base of support (BOS) [6], or by adding a cognitive task. For strength training, it is recommended in literature that the muscles are sufficiently challenged by increasing the intensity of the exercises or the number of repetitions, so that the muscles will fatigue [18]. The American College of Sports Medicine defined the threshold for hypertrophy and strength gains to be 60% of the one-repetition maximum [19]. However, exercises with external weights are unpractical in VR training, which is often performed at home. Recent research showed that strength exercises at low loads, but with high velocities, can induce muscle activations comparable to training with high loads [20]. Furthermore, these low-load exercises also seem to induce benefits for strength and balance in older adults [21]. Finally, ongoing participation in the training program is recommended to prevent fading of the gained benefits [6]. A study that analysed the challenge of balance provided by off-the-shelf games showed that balance is challenged to a varying extent, but that ample room for improvements is left. Moreover, it was found that adaptation to or learning the game, as trials advanced, resulted in a decreasing challenge in some games [22, 23]. From the analysis of muscle activity in seven off-the shelf games, it was concluded that overall muscle activation was low and that longer periods of muscle activation were scarce [24]. Only the games that required faster movements elicited some muscle activity that seemed challenging enough to be considered as a training impulse [24].

The motivational pull of exergame balance training with off-the-shelf games, was assessed in older adults and results showed that playing exergames can lead to strong intrinsic motivation [25]. Especially games that provide positive feedback resulted in high intrinsic motivation. Furthermore, physically active games containing variation seemed to be the preferred game mechanics [25].

Based on the above summarized recommendations for balance training (e.g. sufficiently challenging balance tasks and strength exercises that lead to muscle fatigue), an exergame package for balance training for older adults was developed [2, 4, 6]. The aim of the current study was to evaluate whether the novel set of exergames (called Virbal), which are controlled with off-the-toy-shelf technologies, are feasible and well-suited from a content perspective for balance training in elderly. The novel games were evaluated to see whether they were more challenging in terms of balance movements and muscle activity than existing off-the shelf games. Furthermore, the novel exergames were evaluated on how motivating they are for older adults. Games were compared regarding the challenge imposed to balance in terms of magnitude of center of mass (COM) displacements and regarding the muscle activation elicited in terms of intensity and duration of muscle activation. Motivation was evaluated using questionnaires on motivation.

## Methods

Sixteen healthy older adults, who reported to have no physical or cognitive diseases and could stand for at least 20 min, were recruited by distributing flyers at sports facilities and other social activities for older adults around Leuven (Table 1). All participants were older than 65, lived independently and scored above the inclusion threshold of 26 on the Mini Mental State Examination (MMSE) [26]. All participants signed a written informed consent, in accordance with the declaration of Helsinki. The local ethics committee (Commissie Medische Ethiek K.U. Leuven) approved the study.

**Table 1:** participants characteristics

Number	16
Age (years)	69.13 (2.6)
Gender (f/m)	9/7
Height (cm)	168.6 (9.5)
Weight (kg)	67.06 (9.6)
MMSE	29.06 (0.9)

Values are displayed as mean (SD)

### Games

Six exergame applications were tested. Four (Wasps, Slingshot, Garage and Fishing) were part of *Virbal,* a novel exergame training package focusing on balance, developed in dFlow (Motekforcelink, Amsterdam, Netherlands). The remaining two were off-the-shelf games: *Adventure*, Kinect Adventures (Ubisoft, Rennes, France) and *Kinski,* Kinect sports season 2 (Microsoft Studios, Redmond, WA, US)*.* Adventure and Kinski were included in this study because they showed to be the games that previously had most induced muscle activity and weight shifts [22, 24].

**Fig. 1:**
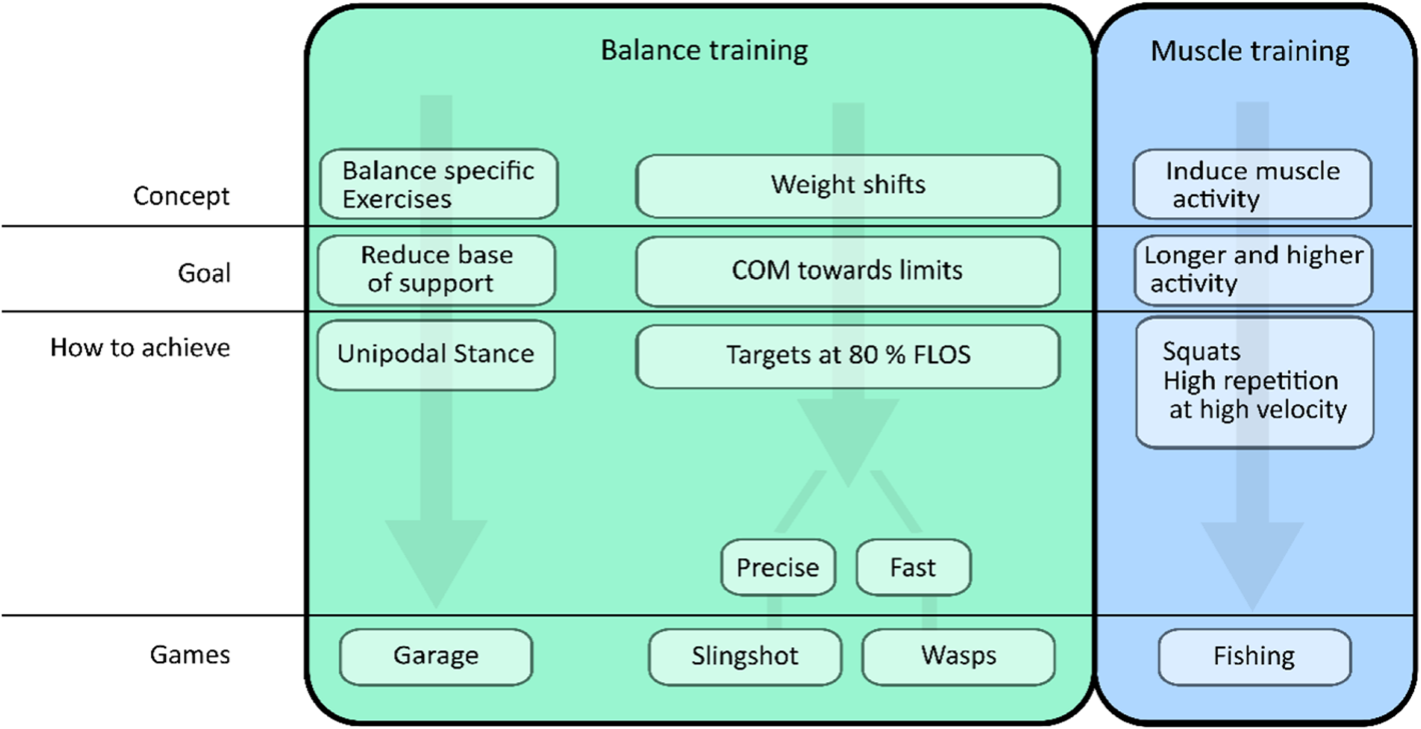
An overview of the concepts used in the development of the Virbal mini games

Based on the recommendations for balance and strength training, obtained from the literature [2, 6, 18, 27] and our previous work in training using exergames [22, 24, 25], several concepts to be addressed in exergames balance training applications were proposed. In Fig. 1, an overview of the translation of these concepts into four mini games is provided: the games Wasps and Slingshot were developed to elicit challenging weight shifts. Of those two games, Wasps focuses on speed, while Slingshot elicits additional cognitive load while challenging balance. Therefore, these games and the Kinski game were included for the COM analysis, whereas muscle activity was studied in the games Adventure, Fishing and Garage. The Garage game was mainly designed to include the balance-training concept of reducing the BOS by including single leg stance. Even though muscle strength was not the primary target for this game, single leg stance likely requires substantial muscle activation for stability. The Virbal games and the Functional Limits of Stability (FLOS) task were all controlled using the Kinect sensor, X-box 360 (Microsoft, USA). More information on the different games can be found in the appendix and in Fig. 7.

### Weight shifts

During game play, 3D-kinematics were captured using seven MX-T20 opto-electronical cameras (Vicon, Oxford Metrics, UK) at 100 Hz. Based on these 3D-kinematics,full-body COM was calculated in Matlab (Math Works, Natick MA, USA). The COM was calculated based on a 4 clusters, 31-markers, 15-segments full-body linked segment model [28], such that each segment was tracked by at least three markers or a cluster. During the game, the COM was calculated online using a simple model (consisting of a three-segment model, including two legs and a trunk) and movement data from the Kinect [29]. This online calculated COM was used to control the games.

Weight shifts during the off-the-shelf game Kinski and the novel mini games Slingshot and Wasps were studied based on COM displacements. To quantify their challenge, weight shifts were expressed relative to the FLOS. In the FLOS test, participants receive visual feedback on their COM and are instructed to move their COM as far as possible into eight directions [22]. This test represents the individual, functional limits of stability, as opposed to theoretical limits of stability based on anthropometry. The FLOS values are used as setting for the balance components in the Virbal game. In addition, the speed of COM displacements was assessed. When moving at high speed, it is more difficult to bring the COM to the limits of stability. By calculating maximal speed of COM movements in the different games, correction for this possibly confounding factor can be made, if needed.

### Muscle activity

Muscle activity was measured using surface electromyography (EMG), with silver-silver chloride, pre-gelled bipolar electrodes (Ambu Blue Sensor, Ballerup, DK), over five lower limb muscles previously shown to be important for balance and fall prevention [4, 30, 31]; m. Biceps Femoris (BF), m. Vastus lateralis (VL), m. Vastus medialis (VM), m. Soleus (Sol) and m. Gluteus medius (GluM). All electrodes were placed according to SENIAM guidelines [32]. The circular electrodes were trimmed to allow 2 cm inter electrode distance and were connected to an eight-channel wireless EMG system (Aurion, Zero-wire, IT), and signals were acquired in Nexus (Vicon, Oxford Metrics, UK) at 1000 Hz. All processing was performed in Matlab (Math Works, Natick MA, USA). EMG signals from both experimental and maximal voluntary contractions (MVC) trials were high-pass filtered at 20 Hz, using a 3rd order high-pass Butterworth filter, before being rectified and smoothed using a moving average technique with a time window of 100 ms. The processed signals from the experimental trials were then normalized to maximum values obtained during the MVCs [24].

Muscle activity was analyzed during the off-the-shelf game Adventure and the novel games Fishing and Garage. A short description of the post-processing methods used to quantify muscular challenge is described in more details elsewhere [24]. In short, the normalized EMG signals were divided in blocks of 200 ms. Based on the average activation in each block, each episode was then categorized in one of four activation zones, < 40%, 40–60%, 60–80 and > 80% MVC. The number of blocks in each zone was counted and total time spent in each zone was calculated and normalized to the duration of the game, to express the Time in Zone (TIZ). A high number of consecutive blocks of muscle activation is seen as representative of higher metabolic stress, a crucial factor in strength gain following exercise at lower intensities [33]. Therefore, the maximal number of consecutive 200 ms blocks (MCB), separated by no more than 3 seconds, of < 40% MVC EMG activity was quantified.

### Protocol

Before participants engaged in the games, EMG electrodes were placed. For practical reasons, we chose to measure muscle activity unilaterally on the left leg. Muscle activity during isometric MVCs was obtained according to the SENIAM guidelines. Thereafter, the markers were placed and participants’ FLOS were determined. The FLOS values are used as setting for the balance components in the Virbal game. Participants then played the games in a randomized order. Randomization was performed on the level of the main games and on the level of the subgames for the Virbal game. For the main games, all combinations of randomization were performed by at least one subject. The duration of the games differed between games. However, mean duration for the subgames of Virbal and the Adventure game was 2 min, for the Kinski game was this around 1 min. Each game was played three times (trials), after which, the participants were asked to sit down and take a rest for approximately 5 min during which they were asked to fill in the Intrinsic Motivation Inventory (IMI) questionnaire [34].

### Motivation

The questions of the IMI questionnaire are categorized into seven different subscales from which the relevant subscales were included in the study [34]: *enjoyment, competence, effort, value,* and *tension,* of which *tension* is a negative trait and enjoyment can be considered as a self-report of intrinsic motivation [34]. For all subscales, Cronbach’s alphas are relatively high (Table 2), indicating internal consistency between the items that constitute each subscale. However, the subscale effort and tension show less consistency. It is also shown to be a temporal stable measurement tool [35]. The IMI was set up in Access (Microsoft, Redmond, USA), so that the order of the questions was randomized.

**Table 2:** Cronbach’s alpha for each subscale

IMI subscale	Cronbach’s Alpha
Enjoyment	0.925
Competence	0.923
Effort	0.548
Tension	0.681
Value	0.899

### Statistics

No previous effect sizes were available to estimate power and sample size. Since our main focus was to test the proof of concept and feasibility of the novel exergames (capable of elucidating challenging movements for muscle and balance), only a limited sample size of 16 subjects were recruited. This is in analogy of previous biomechanical studies on exergames [36, 37]. Generalized Estimated Equations (GEE), tested the differences between games and trials on the COM displacements expressed as a percentage of FLOS. Post-hoc pairwise comparisons were performed using Bonferroni corrections. Furthermore, GEEs were used to test differences between games and on TIZ and MCB for all five muscles, with trial number as a covariate. Post-hoc pairwise comparisons were done using Bonferroni corrections. IMI scores were compared across the different games using Friedman’s ranked ANOVAs. All statistical analysis were performed in IBM SPSS Statistics Version 21.0. Differences at the level *P* < 0.05 were considered statistically significant. A value that is more than 1.5 times the interquartile range away from the top or the bottom of the boxplot was considered and was shown in the figures as an outlier.

## Results

### Weight shifts

**Fig. 2:**
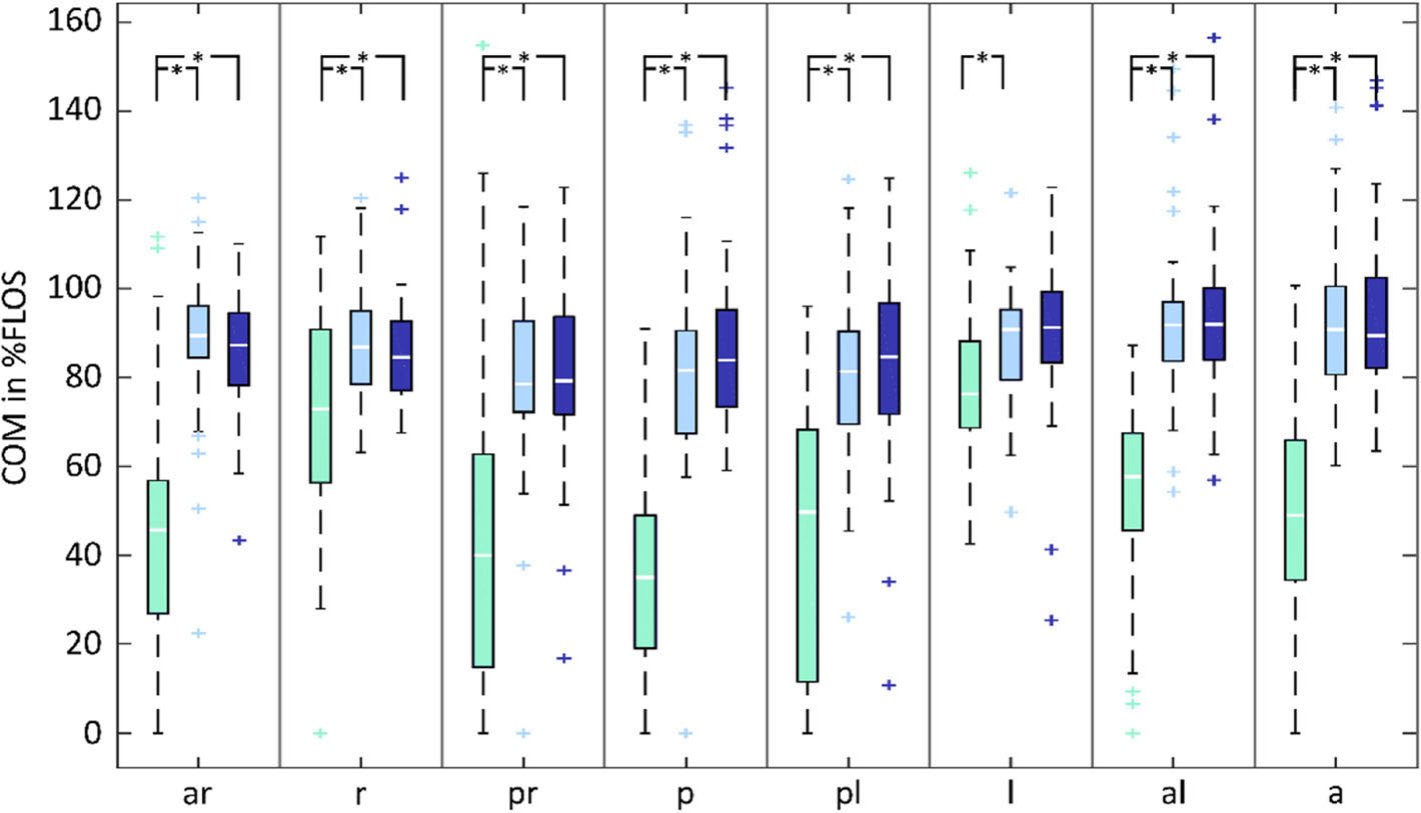
COM displacements expressed as %FLOS in all directions. Slingshot in dark blue, Wasps in light blue and Kinski in green

The COM displacements expressed as a percentage of FLOS are represented in Fig. 2. GEE model effects for the COM displacements as %FLOS for all eight directions are shown in Table 3. Wald Chi Squared values and degrees of freedom are presented in Table 1 of the appendix. For all directions, a significant effect of game was found, no trial effects were seen and only for the L-direction a game x trial effect was found. The post-hoc analyses, after Bonferroni corrections, clarify that for all directions Wasps elicited significantly larger COM displacements than Kinski (Fig. 2). Similarly, Slingshot elicited larger COM displacements than Kinski in all directions, except for the left direction (Fig. 2). For Slingshot, the left direction was only significantly different from Kinski during the third trial, where the elicited COM excursion was significantly larger than in trial one. No significant differences were found between Wasps and Slingshot for any direction. Finally, even though peak COM velocity was higher in Kinski (0.48 m/s +/− .09) and Wasps (0.43 m/s +/− .08) compared to Slingshot (0.28 m/s +/− .04), no significant differences were observed.

**Table 3:** *P*-values for the null hypothesis tests from the GEE results on COM displacements as %FLOS

Direction	Game	Trial	Game x trial
AR	**<.001**	.370	.246
R	**<.001**	.853	.299
PR	**<.001**	.759	.166
P	**<.001**	.804	.167
PL	**<.001**	.770	.513
L	**<.001**	.123	**.033**
AL	**<.001**	.973	.320
A	**<.001**	.417	.754

*Abbreviations for the directions*: *AR* Anterior right, *R* Right, *PR* Posterior right, *P* Posterior, *PL* Posterior left, *L* Left, *AL* Anterior left, *A* Anterior. Significant values are shown in bold

Median values are indicated with a horizontal line, the box ranges from the 1st to the 3rd quartile. Whiskers indicate the range of the data. Significant game effects (*) and outliers (+) are indicated. The blue-dotted line represents the set target distance for Wasps and Slingshot.

Abbreviations for the directions: ar = anterior right, r = right, pr = posterior right, p = posterior, pl = posterior left, l = left, al = anterior left, a = anterior.

### Muscle activity

### Percentage TIZ

**Fig. 3:**
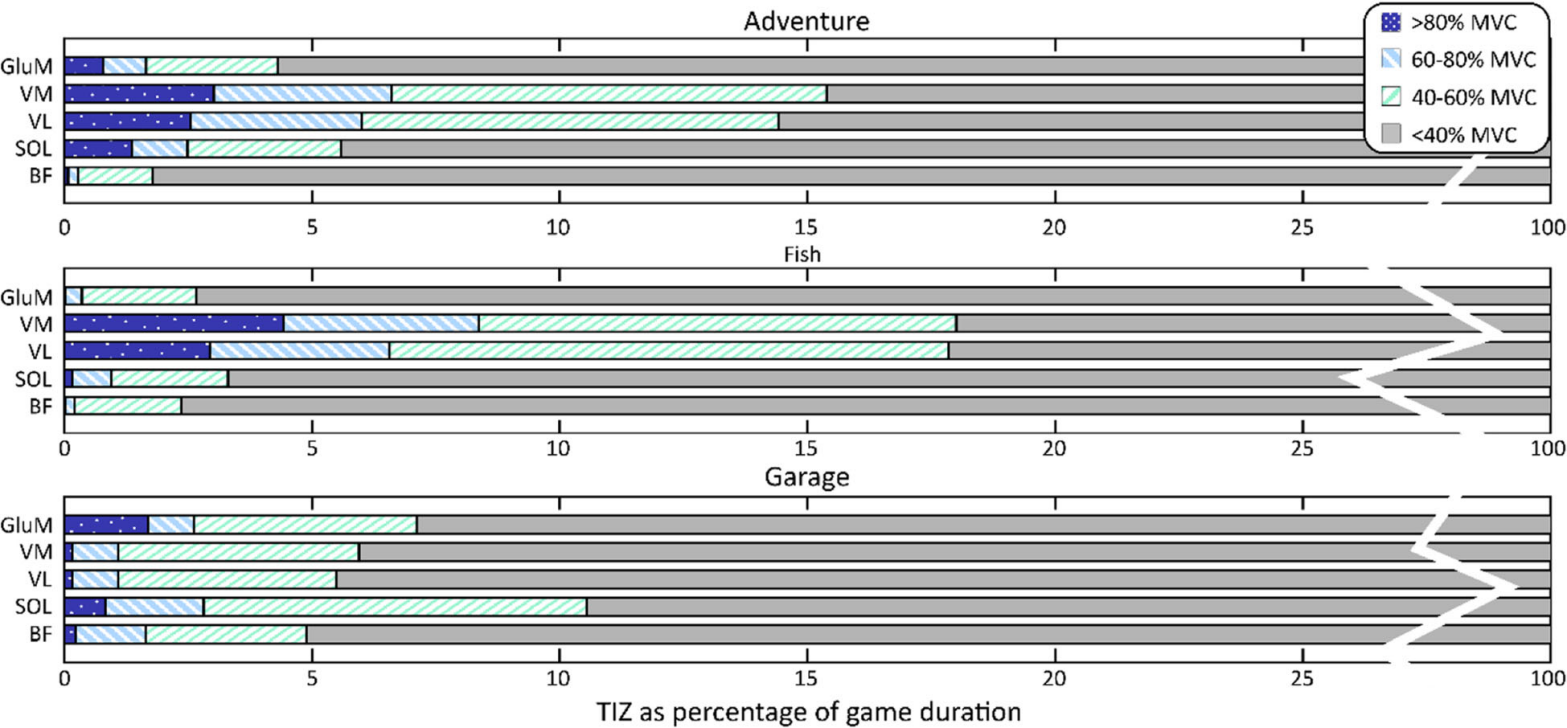
Distribution of muscle activity over different zones for three games as percentage of total game time. Activity higher than 80% of MVC is colored in dark blue, activity between 60 and 80% is represented in light blue stripes, between 40 and 60% in green stripes and below 40% of MVC is grey

**Fig. 4:**
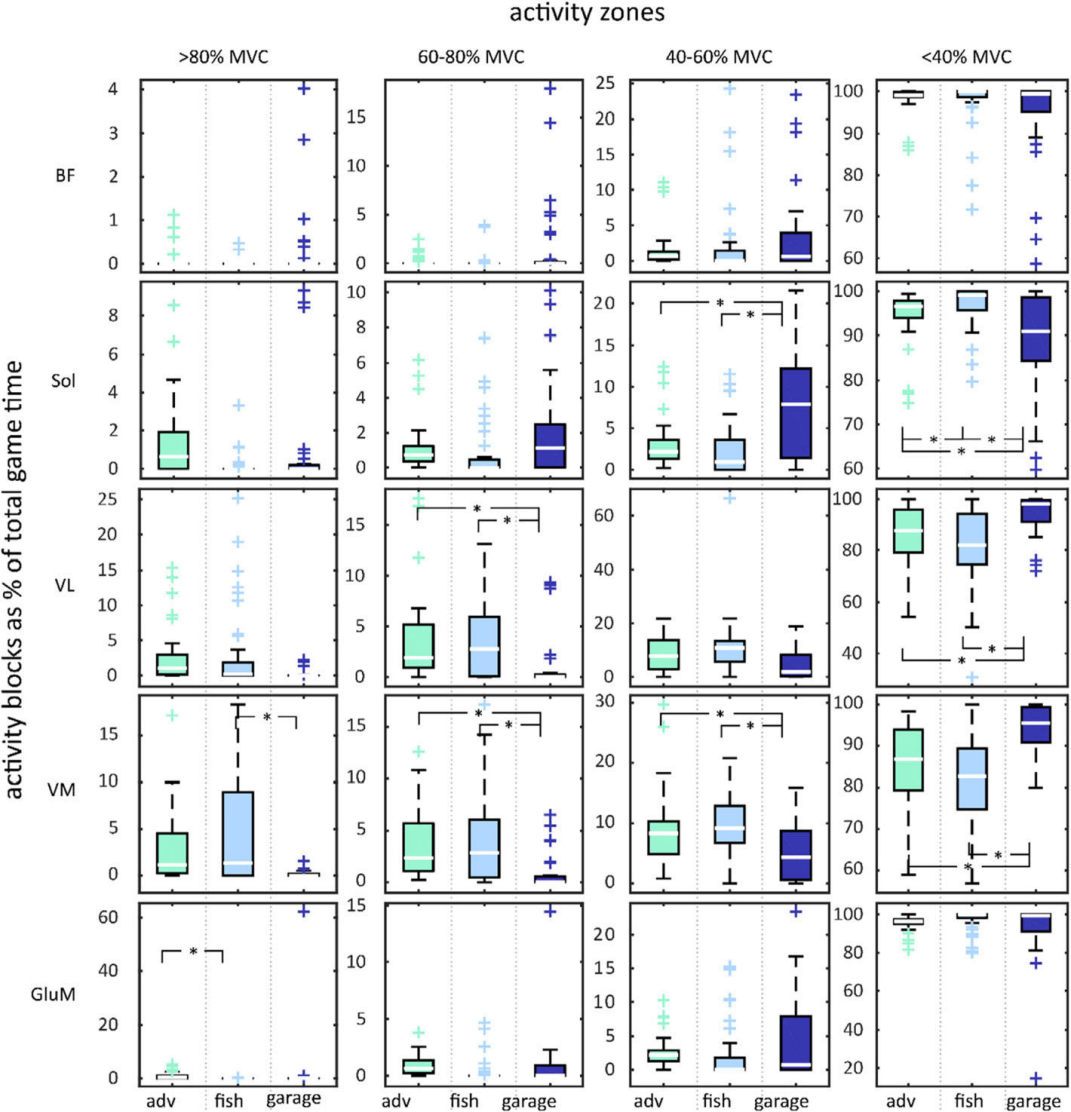
Post-hoc comparisons of the TIZ between games. Garage in dark blue, Fishing in light blue and Adventure in green

Model effects of the different games on muscle activity are represented in Table 4. Wald Chi squared values and degrees of freedom can be found in Table 2 in the appendix Graphical representations of the duration of muscle activity in different zones for the three games are provided in Fig. 3 and post hoc comparisons are represented in Fig. 4. No trial effects were found for any of the muscles or games.

**Table 4:** GEE results for both muscle activity measures (MCB and TIZ) in the Fishing, Garage and Adventure game

Muscle	Time in Zone (TIZ)				MCB
	< 40%	40–60%	60%80%	> 80%	
BF	**.041**	.061	.072	.614	**<.001**
Sol	**.001**	**.004**	.156	.071	**<.001**
VL	**.001**	.077	**.004**	0.101	**<.001**
VM	**<.001**	**.002**	**.007**	**.003**	**<.001**
GluM	.957	.136	.833	**.001**	0.131

Significant values are shown in bold

For the **BF** muscle, a significant effect of game was only found in the < 40% MVC zone. Post hoc comparisons reveal that there was a trend indicating longer TIZ in the lowest activity zone in the Garage game compared to Fishing.

For the **Sol** muscle, significant game effects were also found. First, a game effect was found in the 40–60 zone. Post hoc comparisons revealed that Garage elicited longer TIZ than Adventure and Fishing. Additionally, a significant effect of the factor game was found for the < 40% category. Post hoc analysis revealed shorter time in the lowest activity zone in the Garage game compared to both Adventure and Fishing. Furthermore, a slightly shorter time in the lowest zone was found during Adventure compared to Fishing.

For the **VL** muscle, TIZ in the 60–80% MVC category was significantly affected by game. Post hoc analysis revealed that Fishing and Adventure resulted in longer TIZ in the 60–80% zone than Garage. Furthermore, TIZ in the < 40% category was affected by game. Post hoc analysis revealed that Adventure and Fishing both resulted in shorter TIZ for the lowest activity zone than Garage.

For the **VM** muscle, the TIZ for each category was affected by the factor game. Post hoc results show that for the > 80% MVC category Fishing showed longer TIZ than Garage. In the 60–80% category both Fishing and Adventure showed longer TIZ than Garage. In 40–60% category, both Fishing and Adventure showed longer TIZ than Garage. Consequently, both Adventure and Fishing showed less TIZ in the lowest category than Garage.

Finally, for the **GluM** muscle there was an effect of game on the TIZ in the highest activation zone. The GluM showed significantly longer TIZ in the > 80% MVC category during Adventure than during Fishing.

Each row represents a different muscle, columns represent activity zones. Median values are indicated with a horizontal line, the box ranges from the 1st to the 3rd quartile. Whiskers indicate the range of the data. Significant game effects (*) and outliers (+) are indicated.

### Maximal consecutive blocks

**Fig. 5:**
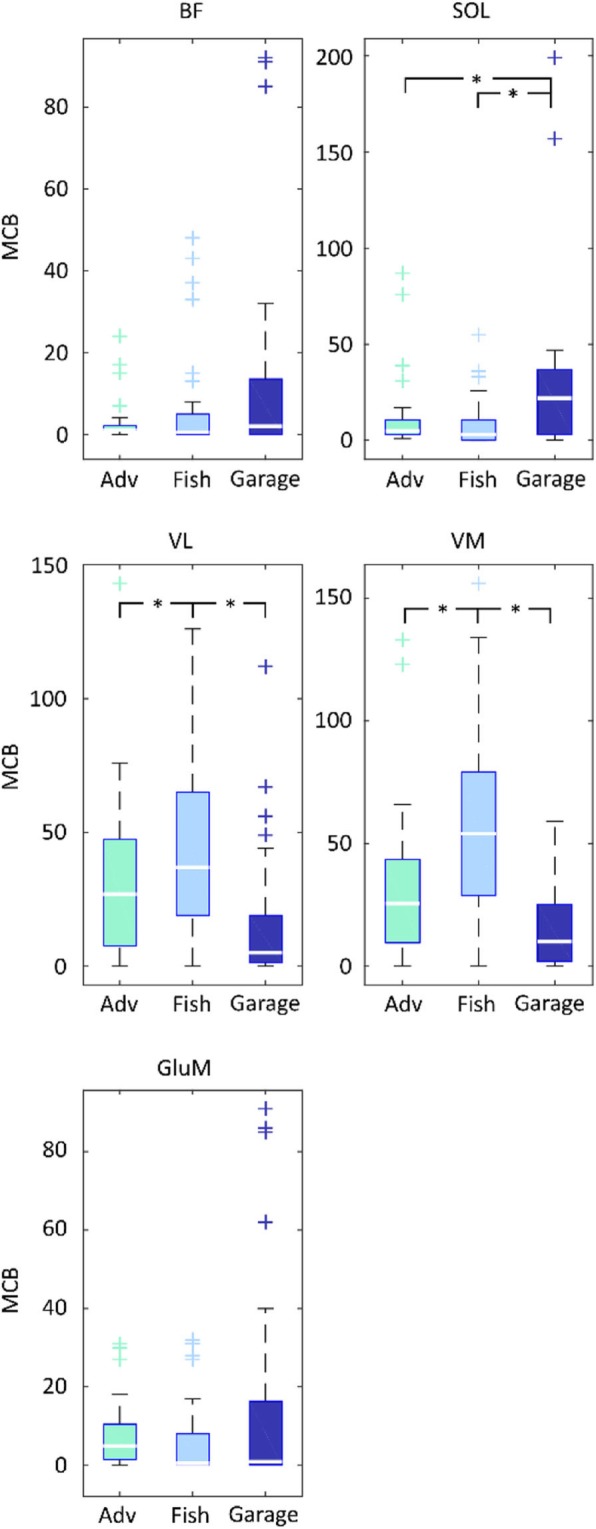
Post-hoc comparisons of the MCB measure between games

The differences between games for the MCB measure are shown in Fig. 5. There was a main effect of game for **BF**, however no significant difference was found in the post hoc tests after Bonferroni corrections. For **Sol**, the Garage game elicited a significantly higher number of MCB compared to the Adventure and Fishing games. For both **VL** and **VM**, the number of MCB was higher in Fishing than in Adventure and Garage. No significant differences in MCB were found for **GluM**. No trial effects were found for any of the muscles or games.

Median values are indicated with a horizontal line, the box ranges from the 1st to the 3rd quartile. Whiskers indicate the range of the data. Significant game effects (*) and outliers (+) are indicated. Slingshot in dark blue, Wasps in light blue and Kinski in green.

### Intrinsic motivation

**Fig. 6:**
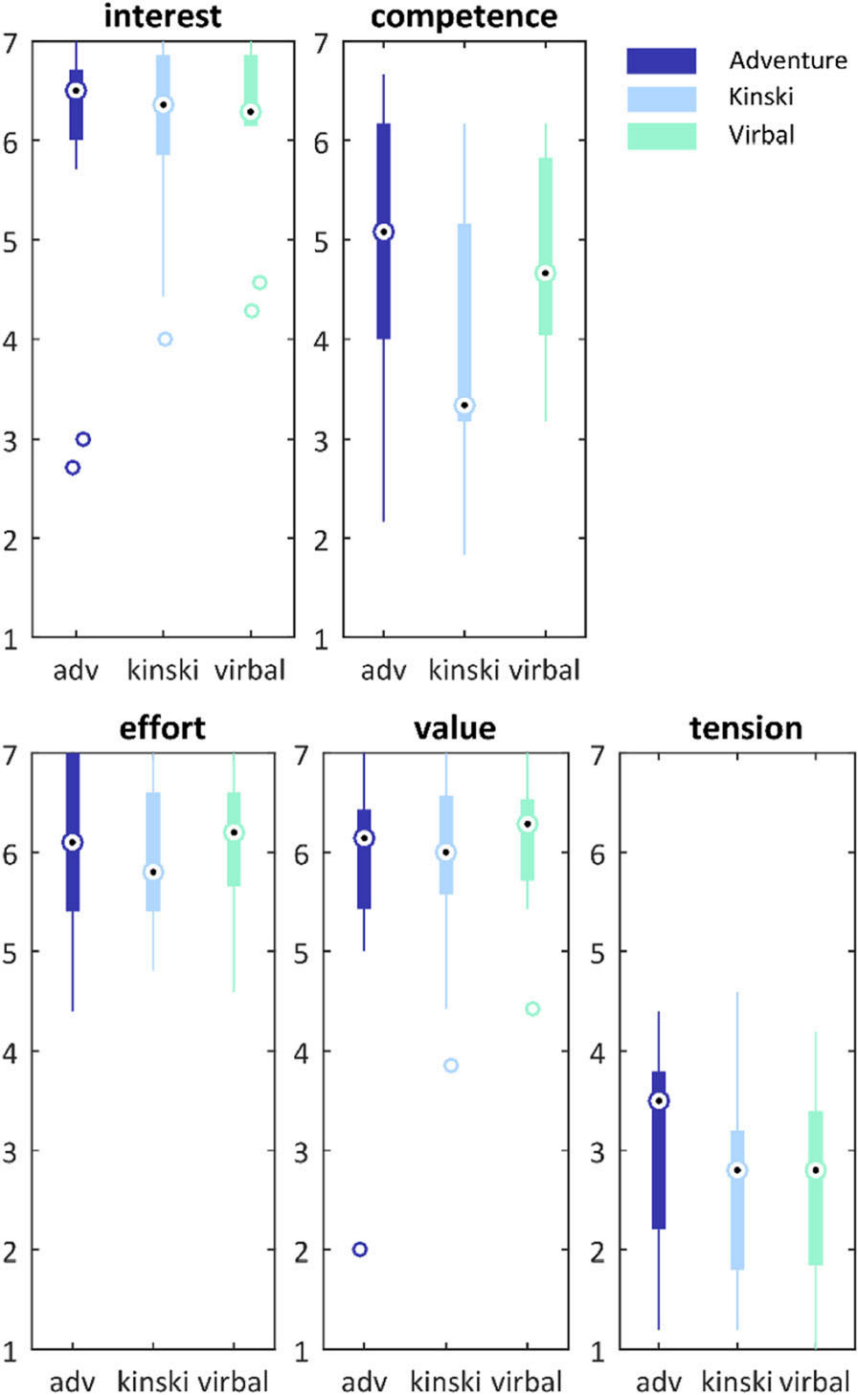
The descriptive statistics of the IMI data. Slingshot in dark blue, Wasps in light blue and Kinski in green. Higher scores represent a better evaluation of the game, except for the subscale tension, which represents a negative characteristic. Medians are marked as a circle, the box ranges from the 1st to the 3rd quarter. Whiskers indicate the data range and outliers are marked with circles

Results from the IMI questionnaire (Fig. 6) show that the Virbal games lead to similar and high levels of motivation as the off-the-shelf games. No significant differences in ranking on any of the subscales was found between games: *interest* X^2^ (2) = 0.520, *p* = .771; *competence* X^2^ (2) = 4.353, *p* = .113; *effort* X^2^ (2) = .585, *p* = .746; *value* X^2^ (2) = 2.122, *p* = .346; *tension* X^2^ (2) = .051, *p* = .975.

## Discussion

The aim of this study was to evaluate the potential of the novel balance training application using exergames for balance training in healthy older adults in terms of COM displacements (weight shifts), muscle activity and intrinsic motivation. These games were compared to commercially available games that were previously found to be motivating and somewhat challenging with respect to balance and muscle activation [22, 24].

### Weight shifts

Compared to the other VR games, the Virbal game provokes a higher and consistent challenge to balance in all directions, which should make it more effective for balance training. As previously mentioned, sufficiently challenging weight shifts as measured by COM displacements is a requirement for balance improvement in older adults [38]. From Fig. 2, it can be seen that the difficulty of the game was successfully set to the skill level of the participants, namely above 80% of their maximum and with a small (interquartile) range of data. This should make the Virbal game more effective for balance training compared to the Kinski game (ski-slalom), since the latter one mainly focuses on ML-movements and has a lack of anterior posterior (AP) induced movements. Although the range and interquartile ranges of the data were small, some outliers can be observed in Fig. 2. For the Virbal game, negative outliers were probably due to a technical weakness of the Kinect to measure depth [39, 40]. The outliers seen in the Kinski game may reflect differences in strategy to play the game.

Using simple off the shelf technology, we were able to elicit continuous challenging weight shifts adapted to the individual’s capabilities in the Virbal game by using adequate parameters to control the game. Previously, systematic reviews report varying results of exergames on balance compared to traditional training. However, VR games have the advantage that they can be played at home with only limited equipment. Previous research on exergames showed that the challenge as measured by weight shifts can quickly diminishes over trials, because participants tend to adopt more efficient movement strategies or find tricks to score points more easily with less movement [22]. Such trial effects were not observed in this study. In contrast, the trial effect that for the Slingshot game indicated a positive progression of challenge, with the third trial eliciting larger COM displacements than the first trial. Therefore, by defining weight shifts set at 80% of the obtained FLOS, we were able to ensure an appropriate challenge.

### Muscle activity

The games in the study did not induce high muscle activity, despite our effort to include dynamic exercises in the new game Fishing. However, appropriate metabolic stimulation was confirmed by inducing sufficiently long periods of low intensity. The Fishing game did not elicit longer TIZ for the higher activity levels than Adventure. This indicates that inducing higher-level muscle activity consistently in unloaded strength exercises is difficult. Our results correspond with previous studies, showing that high intensity muscle activity is not observed in training using exergames [24]. However, it has been shown that, in older adults, also strength training at lower intensities can lead to hypertrophy and increases in strength, as long as sufficient repetitions are performed [18]. Exercises at low intensities have the advantage of potentially being more suitable for elderly compared to those at high intensities (e.g. traditional strength training) since these exercises are more accessible (e.g. can be performed without equipment) and pleasant to perform. In the novel games, mechanics that encourage the performance of a high number of repetitions per set were incorporated. The maximal Consecutive Blocks (MCB) better represents this parameter. By adapting the task, training effects, in terms of higher numbers of MCB’s, were adequately directed to the target muscle (VL and VM through squatting and single leg stance for the soleus). However, overall, the number of MCB remains low in most games and a wide variation over participants was performed (Fig. 5).

### Intrinsic motivation

**Fig. 7:**
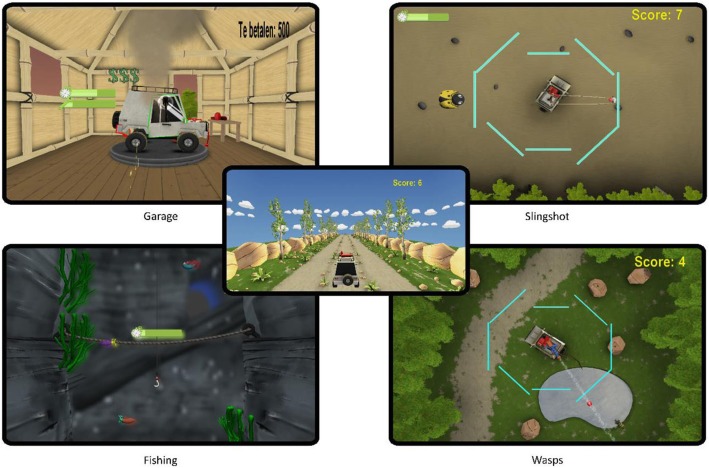
Screenshots of the mini games of the Virbal game. The overarching game is presented in the middle. From this overarching game different mini games are chosen

The intrinsic motivation in all games tested was relatively high on all subscales, suggesting that our sample of older healthy subjects can be intrinsically motivated to participate in exergame training. Motivation is an important feature, considering the high dropout rates in widespread home-based exercise programs for older adults and the fact that a high dose of continued balance training is recommended in literature, to improve functional balance in healthy older adults [33, 38]. To increase intrinsic motivation, it was shown previously that variation and physical activity are important attributes in exergame training for older adults [25]. Therefore, variation in the novel exergame training package was incorporated by creating an overarching game from which several mini games were started. Each mini game aims to challenge a specific aspect of balance and incorporates different game mechanics (see appendix and Fig. 7). However, no significant differences in IMI scores were found between the three different games, possibly due to a ceiling effect in the motivation of participants.

### Limitations

Participants were recruited around sports facilities and active social events for older adults, resulting in a relatively active and fit sample. We did not control for level of physical activity, which can potentially bias the results. Participants with very low physical activity level and low performance level might perform the task more cautiously. In future, the Virbal game should also be tested in a population of older adults that have a high risk of falling to assess its influence on balance. Furthermore, all included participants volunteered to participate in this experiment, resulting in a group of subjects that likely holds positive views towards using exergames technologies. This selection bias might have influenced the high IMI scores. On the other hand, it also shows that there are older adults that show an interest in using exergames.

Experiments were conducted in a carefully controlled laboratory setting. This might have caused participants to behave differently than they would in a home setting. Moreover, our lab, which is specifically set up for motion analysis, presents an ideal environment for sensors such as the Kinect sensor. Further studies are needed to evaluate the performance of the player and hardware in home environments.

Although this study shows that balance training using exergames can be optimized to elicit more challenging movements, as measured by weight shifts and muscle activity, it remains unknown how much challenge in terms of weight shifts and muscle activity is exactly needed to ensure improvements in balance, strength and eventually reduce fall risk. Exergame training might prove to be an effective training form to gain more insight into the required parameters for balance training. This will have to be further explored in longitudinal trials that apply exergame training interventions, of which the intensity is thoroughly defined. Furthermore, the different games had different durations, which make it harder to compare. The duration of the Kinski game and the Adventure game were also depending on the performances of the player. In future, the time of the different games should be controlled to avoid overtraining or on the opposite not enough training.

Lastly, it is important to note that other aspects of training such as for example incorporation of challenging cognitive dual tasks may influence the effectiveness of exergame balance training. However, cognitive load as well as other potential confounding factors on the performance of VR games were not evaluated and were outside the scope of this study.

## Conclusion

A new exergame, named Virbal, was tested on feasibility and showed that the challenge in exergames controlled with affordable, off-the-shelf controllers can indeed be improved when the control algorithms are carefully developed to match the desired challenges. By adapting the game settings to the performance on the FLOS task, the challenge induced by weight shifts was successfully increased and set relative to the personal capacities. However, even though the provocation of muscle activity was improved by eliciting longer bouts of sustained activity, it remained difficult to elicit high muscle activity in the unloaded exergame training. In future studies, the balance effects of long-term training using this new game in older adults should be assessed in a randomized controlled intervention study. Further, training programs using exergames that are optimized to elicit challenging weight shifts and muscle activity should be further studied in longitudinal interventions. These interventions studies should uncover the effects of optimized exergame training on balance, muscle performance and eventually fall risk in older adults.

## Data Availability

The datasets used and/or analyzed during the current study are available from the corresponding author on reasonable request.

## Appendix

A brief description of the mini-games. This appendix give a short description of the mini-games that are included in the novel exergame called Virbal

### A brief description of the mini games in the custom-made game (Virbal)

In a study addressing the user experience in exergame balance training, the importance to incorporate variation and provide a good balance between low and high speed was shown (De Vries 2018). Therefore, one overarching game was developed that provides rest between the mini games, in which the different balance training concepts are addressed (Fig. 1). The decision was made to include playful elements, that would increase fun and motivation, but also recognizable elements that would improve the experience of realism. Accordingly, three mood boards were created, with different levels of graphical details, from cartoony to realistic. The mood boards were shown to a small panel of older adults, who showed a preference for the less detailed, cartoony style. A screenshot of the final graphics is included in Fig. 7.

In the **Garage** game the concept of specific balance exercises such as reducing the base of support (BOS) was addressed (Fig. 1). Therefore this game was controlled by knee raises. The car, which was used in the overarching game would show up in a garage, with several technical problems. The player was given 3 min to repair as many broken items, which reduced the final cost at the garage. To repare an item, the player first had to select this item by raising the knee until the car was rotated such that the item was selected. This required single leg stance for both legs, e.g. lifting the left or right leg would make the car rotate in the left or right direction, respectively*.* Then the player had to take steps in place, if the knee was raised sufficiently (25% of the upper leg length) the reparation would progress.

In the **Slingshot** game, the main concept was to induce challenging weightshifts and a dualtasking component. Hence, movements in this game were controlled by center of mass (COM) displacements. The COM was calculated using a simple three segment model (two legs and an upper body), based on the formulas and anthropometrics as presented by Winter et al. [2]. The player had to feed the correct colorred berries to ladybugs. Berries had to be collected and loaded into a slingshot, the slingshot had to be precisely aimed at the lady bug and the berry would be shot towards the ladybug only if the player would move his/her COM at least at 80% of the FLOS for that direction.

In the **Wasps** game, the concept of weight shifting was also addressed, but this time the focus was on fast instead of precise weight shifts. Movements were again controlled by COM displacements. In this game, the player would find himself at a wasp infested location. To keep the wasps from stinging a water gun could be used to get rid of the wasps. The water gun provided a continous stream as long as the player would move their COM at least at 80% of the FLOS for that direction. Therefore, precision was less important, but due to the increasing appearance of new wasps, the speed was more important.

Finally, in the **Fishing** game the aim was to incorporate movements that elicited muscular challenges. The objective in this game was to catch as many fish as possible. To do so, the player had to lower the fishing lure into deeper waters by performing squats. Squats were monitored through the Kinect camera by following the height of the hips relative to the knees. The intensity of this game was adjusted to different skill levels by varrying the number of repetitions. If the player was fast enough in the first section, he/she would be awarded extra time to continue progressing to the next section. When the time ran out before a section was completed, the lure went up and the player had to catch the fish, by performing small movements of the COM. The catching phase was intended as a recovery phase from the squats, not to induce challenging weight shifts. After each game the player returned to the overarching game in which they drove their truck to the next mini game.

#### A brief description of the off-the-shelf games

Adventure (Kinect): We selected the Reflex Ridge mini game from the Kinect Adventures package (Ubisoft, Rennes, France). In this game, the player stands on a platform that moves along a set of rails. Various objects have to be avoided by dodging, jumping and taking side steps. Movements are tracked with the xbox Kinect sensor.

Kinski (Kinect): A mini game from the Kinect sports season 2 (Microsoft Studios, Redmond, WA, US) package. The player has to ski as quickly as possible down a slalom course, however missed slalom gates will result in penalty time. Extra speed can be gained by jumping at the right moments. The player controls the avatar by using weight shifts, as captured by the kinect sensor (Microsoft, Redmond, WA, US).

**Table 5:** Wald Chi Squared values and degrees of freedom for COM displacements for game, trial and interaction effect for eight directions

	Game (df = 2)	Trial (df = 2)	GamexTrial (df = 4)
AR	95.750	1.99	5.432
R	16.939	0.318	4.889
PR	27.941	0.552	6.482
P	73.312	0.436	6.458
PL	33.893	0.523	3.272
L	14.993	4.189	10.464
AL	47.716	0.054	4.699
A	54.229	1.751	1.901

**Table 6:** Wald Chi Squared values and degrees of freedom for TIZ and MCB for all muscles

*Game*	TIZ				MCB
Df = 2	> 80%	60–80%	40–60%	< 40%	
BF	0.976	5.272	5.605	6.409	30.280
SOL	5.281	3.716	11.122	13.779	121.342
VL	4.576	11.002	5.122	13.869	15.204
VM	11.718	9.997	12.989	32.058	36.040
GluM	13.793	0.366	3.991	0.088	4.063

## Abbreviations

A: Anterior
AL: Anterior-Left
AP: Anterior-Posterior
AR: Anterior-Right
BF: Biceps Femoris
COM: Center of Mass
FLOS: Functional Limits of Stability
GEE: Generalized Estimated Equations
GluM: Gluteus Medius
IMI: Intrinsic Motivation Inventory
L: Left
MCB: Maximal number of consecutive blocks
ML: Medio-lateral
MMSE: Mini Mental State Examination
MVC: Maximal Voluntary Contraction
P: Posterior
PL: Posterior-Left
PR: Posterior-Right
R: Right
SOL: Soleus
TIZ: Time In Zone
VL: Vastus Lateralis
VM: Vastus Medialis
